# Supplementary material for: Squalamine reverses age-associated changes of firing patterns of myenteric sensory neurons and vagal fibres
Source: Commun Biol. 2024 Jan 10;7:80. doi: 10.1038/s42003-023-05623-2 (PMC10781697; doi:10.1038/s42003-023-05623-2)
Supplement: Supplementary file 2 — Supplementary Information [file 42003_2023_5623_MOESM2_ESM.pdf]

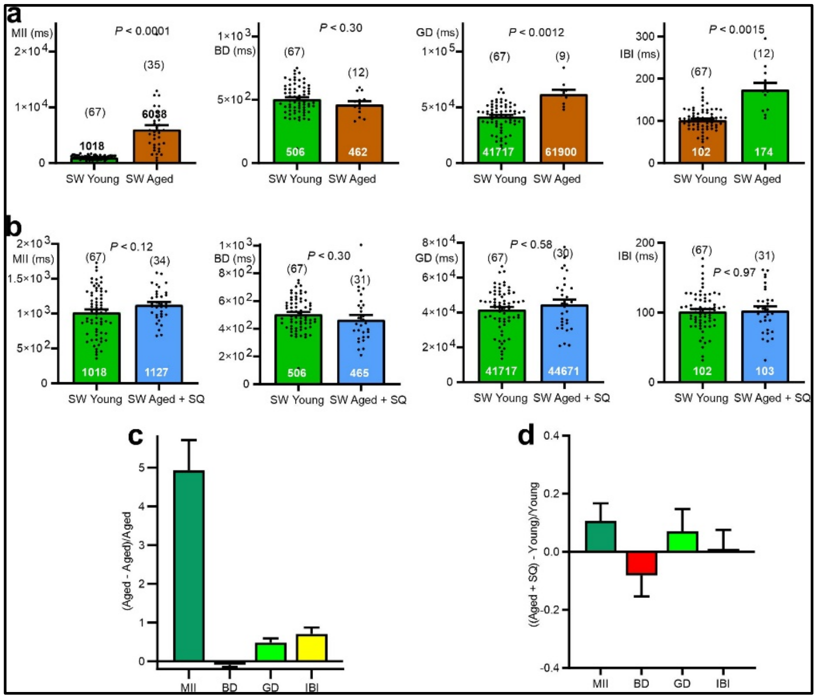

Supplementary figure 1 The ageing code in Swiss Webster (SW) mice. MII, GD, and IBI, but not BD were all increased in aged compared to young single units. a, Dot plots with superimposed bar graphs (mean ± s.e.m.) showing values for all 4 single unit firing parameters. Differences between young vs aged mice for MII, GD and IBI reached statistical significance. b, No statistical difference was discernible for any of 4 firing pattern parameters when young mice luminally perfused with Krebs buffer were compared to aged mice whose lumen was perfused with Krebs containing 30 μM squalamine. c, The ageing code revealed by plotting fractional differences (mean ± s.e.m.) of young vs aged mice for each of the 4 firing parameters. All of parameters except for BD contributed to the code. d, The ageing code was eliminated when the lumen of aged animals was perfused with Krebs with added squalamine and compared to young mice whose jejunal lumen was perfused with Krebs only. Statistics: a,b, Comparisons of parameter means for young vs. aged or young vs. aged + squalamine made using Dunnett's T3 multiple comparisons tests

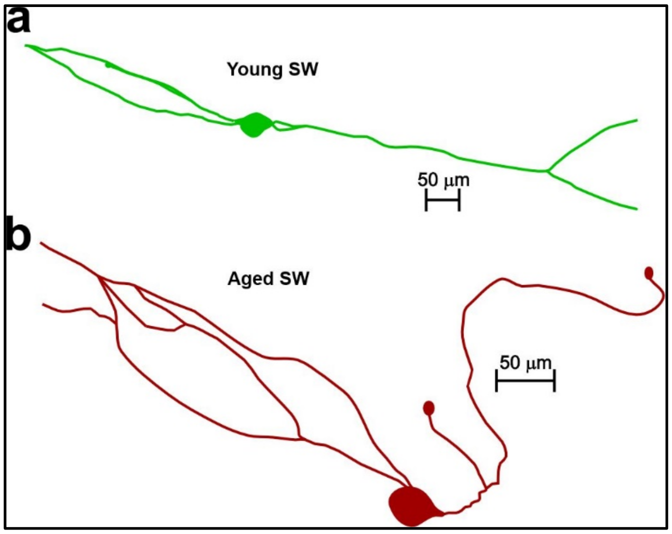

Supplementary figure 2 Shapes of IPANs recorded from myenteric plexuses of Swiss Webster (SW) mice. a,b, Representation of IPAN shapes revealed after intracellular neurobiotin dye filling. IPANs Dogiel type II cell morphology. Some axons terminated in end bulbs (rounded swelling in b), these are swellings where axons were broken during dissection and then have resealed.
